# Movement behavior in adults with sickle cell disease compared to healthy adults: a cross-sectional study

**DOI:** 10.1371/journal.pone.0336932

**Published:** 2026-04-15

**Authors:** Aaron A. Heeren, Eduard J. van Beers, Bart J. Biemond, Myrthe J. van Dijk, Erfan Nur, Charlotte F.J. van Tuijn, Merel A. Timmer

**Affiliations:** 1 Centre for Benign Haematology, Thrombosis and Haemostasis, Van Creveldkliniek, University Medical Centre Utrecht, Utrecht University, Utrecht, The Netherlands; 2 Department of Hematology, Amsterdam UMC, University of Amsterdam, Amsterdam, The Netherlands; 3 Department of Blood Cell Research, Sanquin Research and Landsteiner Laboratory, Amsterdam, The Netherlands; Kintampo Health Research Centre, GHANA

## Abstract

**Background:**

Sickle cell disease (SCD) is one of the most common monogenic disorders in the world. Patients with SCD have chronic hemolytic anemia and experience episodic pain due to vaso-occlusion resulting in, amongst other, musculoskeletal and cardiopulmonary complications. We hypothesize that adults with SCD are less physically active, but limited information is available on the movement behavior of adults with SCD. In the current study, we aimed to evaluate movement behavior in patients with SCD as compared to healthy controls.

**Methods:**

Movement behavior of patients with SCD (≥16 years) was prospectively collected in two University Medical Centers in the Netherlands. Movement behavior was measured for seven consecutive days with an accelerometer (Activ8), distinguishing between lying/non-wear, sitting, walking, running, and biking. Time spent on activities was compared between patients with SCD and healthy adults with a migration background and between HbSS/HbSβ^0^ and HbSC/Hbβ^+^using the Man-Whitney U test. To adjust for multiple testing, season, sex, and age, a linear regression for each activity was performed.

**Results:**

Data of 30 patients with SCD during steady state (median age 32.1 (IQR 25−47)) and 57 healthy adults (median age 37 (30−50)) were analyzed. Patients with SCD walked 0.68 (CI −1.22 to −0.14) h/day less than healthy adults. No differences were identified for other postures and activities. Every point increase in hemoglobin level was associated with 0.26 (CI 0.01 to 0.52) h/day increase in walking duration.

**Conclusion and key findings:**

Patients with SCD appear to be less physically active compared to healthy adults, which is mainly reflected by reduced walking time (around 3500 steps less a day), highlighting the need for tailored physical activity support.

## Introduction

Sickle cell disease (SCD) is an autosomal recessive monogenic genetic disorder of the hemoglobin molecule [1,2]. Patients with SCD have chronic hemolytic anemia due to HbS polymerisations, recurrent vaso-occlusion, a chronic inflammatory state, and an enhanced coagulation activation, resulting in organ damage and a limited life expectancy [3,4]. The most common genotypes of SCD are HbSS, HbSC, HbSβ^+^-thalassemia (HbSβ^+^), and HbSβ^0^-thalassemia (HbSβ^0^) [3,4] Worldwide, it is estimated that 7–9 million patients suffer from SCD, with the highest prevalence in Africa, India, the Middle East and the Mediterranean region [5,6]. SCD prevalence is increasing in Europe and America due to immigration. The estimated prevalence of SCD in the Netherlands is 1250–1500, and approximately 40–60 children with SCD are born each year [7,8].

Patients with SCD experience a range of disease-related complications affecting multiple organs.^9^ Recurrent vaso-occlusive pain episodes (VOE) caused by microvascular obstruction is the most common complication of SCD [9,10].SCD is also characterized by fatigue and dyspnea, often resulting from severe chronic hemolytic anemia [9,10]. Cardiopulmonary complications such as acute chest syndrome (ACS), pulmonary hypertension, and musculoskeletal complications (osteonecrosis) are also common in SCD [9,10]. These complications severely impact daily functioning [9, 11] and reduce quality of life of patients with SCD [12,13].

Significant advances in treatment and prevention of the complications of SCD have increased life expectancy of these patients [14]. As a result, SCD has changed from a life-threatening disease for children to a chronic condition in adulthood. However, the life expectancy for patients with SCD in affluent countries is still approximately 22 years shorter compared to the general population [15]. It is well-established that patients with chronic health conditions demonstrate a less favorable movement behavior [16,17]. Movement behavior consists of sleeping, sedentary behavior (SB), and physical activity (PA) [18]. PA is categorized into light, moderate, and vigorous PA. Patients with chronic health conditions are generally less active and more sedentary. [19]. SCD-related anemia, pain, inflammation and complications such as avascular necrosis, cardiomyopathy, and pulmonary hypertension create a considerable challenge to be physically active for patients with SCD and may contribute to increased sedentary behavior. Cardiopulmonary exercise testing suggest that reduced exercise capacity in patients with SCD is not only associated with anemia but also with deconditioning, suggesting a less favorable movement behavior [20].

A recent systematic review of one randomized controlled trial, one uncontrolled trial, and four cross-sectional studies suggest that low-to-moderate physical exercises are safe to perform in patients with SCD and increases exercise tolerance and decreases chronic inflammation in patients with SCD [21]. Regular low-to-moderate exercise therefore has the potential to improve quality of life, and improve participation in social activities and potentially reduce SCD-related complications and hospitalizations due to VOE [21].

Given the positive effect of being physically active for patients with SCD, it is essential to evaluate their movement behavior. In order to design effective interventions, it is crucial to understand actual movement patterns in adults with SCD using objective measures. To date, only two studies investigated SB and PA in children and adolescents with SCD, which demonstrated lower levels of PA compared to healthy peers [22,23]. The general physical activity in daily live has not been evaluated yet in adult patients with SCD. We hypothesize that adults with SCD are also less active and have a more sedentary lifestyle compared to healthy adults. The primary aim of the current study is to identify differences in movement behavior between adults with SCD and healthy adults. The secondary aim of the study is to evaluate the association between movement behavior and hemoglobin level, presence of avascular osteonecrosis and plasma levels of NTproBNP.

## Methods

### Design and participants

This cross-sectional study was conducted at the department “van Creveldkliniek” of the University Medical Center Utrecht (UMCU), and the department of hematology of the Amsterdam University Medical Center (Amsterdam UMC), between January and May 2022. Movement behavior data of patients with a diagnosis of SCD (≥16 years) were collected prospectively during their routine outpatient check-ups. Patients dependent on a wheelchair, unable to communicate in Dutch or English, pregnant at time of the check-up or who had undergone orthopedic surgery <1 year prior to the check-up were excluded. Patients who had experienced an acute episode of pain within six weeks prior to the check-up or during the measurement period (without a medically determined cause other than a VOC) that required healthcare provider contact were also excluded [24]. To compare movement behavior of patients with SCD with healthy controls, data of healthy adults with a migration background (at least one parent born abroad) from a previous study using identical Activ8 accelerometry protocols, were use [25]. Since SCD is mostly found in sub Saharan African, the Middle-East, India and southern Europe including Turkey, most patients with SCD in the Netherlands have a migration background. The degree of physical activity is known to vary based on by migration background [26,27]. Therefore, patients with SCD were compared to healthy adults with similar migration backgrounds.

### Ethics statement

The study was approved by the Medical Ethics Research Committee of the University Medical Center Utrecht (study number 21/720). Patients with SCD who were eligible and interested in participating provided written informed consent. The reporting of this study conforms to the STROBE statement.

### Outcome measures

Movement behavior was measured using the Activ8 (A8005), a triaxial accelerometer (30x32x10mm, 20 grams) with an internal sampling frequency of 50 Hz [28]. The Activ8 is a validated activity monitor and can distinguish between lying down/non-wear, sitting, standing, walking, running, and biking [29].The Activ8 identifies every 5 seconds new postures or activities (epoch length), whereby every 5 minutes, a summary of different postures/activities is stored. The operating time is at least 30 days, and data can be stored for 60 days [28]. Participants were instructed to wear the Activ8 in the front pocket of their trousers for seven consecutive days, except during bathing, taking a shower, swimming, or sleeping [29]. Healthy controls received the same instructions as the patients with SCD. After handing in the Activ8, the data were collected with the Activ8 Recording Tool and saved as an Excel file. The time spent on activities was reported in hours per day (lying down/non-wear, sitting, standing, and walking) or minutes per day (running and biking). The minimum wear time for data inclusion was 10 hours on at least four days [30]. PA was measured as defined by Tremblay et al., as the combined time spent on walking, running, and biking (hours per day) [18].

Participants characteristics were extracted from medical health records, including age, sex, genotype, hydroxyurea use, number of admissions for VOC/year, hemoglobin (Hb) level, presence of avascular osteonecrosis, plasma levels of NTproBNP and wearing time of Activ8 (number of days and which season of the year). Season (winter/autumn vs summer/spring), sex and age were considered potential confounders [25,31]. Demographic characteristics of healthy adults, including education level, living area and (co)morbidities, were collected through a questionnaire. The definitions of these variables were used as provided by Statistics Netherlands [32].

### Laboratory analyses

Hemoglobin concentration was determined using an automated colorimetric oxyhemoglobin method on a Sysmex XN-9000 analyzer. Genotyping was performed by high-performance liquid chromatography (HPLC) with UV-Vis detection; if a variant was detected, confirmatory testing by DNA analysis or sickling test was performed. NT-proBNP levels were measured using a sandwich immunoassay on a Roche Cobas Pro e801 analyzer.

### Statistical analysis

Statistical analyses were performed with IBM SPSS Statistics, version 25 (Amork, New York, USA) [33]. Descriptive results were reported as means with standard deviations for normally distributed data or as medians with interquartile ranges (IQR) for not normal distributed data. Data normality was assessed on visual inspection and the Shapiro-Wilks test [34] Boxplots were used to detect outliers with the “1.5x interquartile range” rule [35]

Participant characteristics were compared using the chi-square test for categorical variables and the independent t-test (normally distributed data) or the non-parametric Mann-Whitney U test (for not normally distributed data) for continuous variables. Demographic characteristics of the healthy adults with a migration background were compared to statistics of the Dutch population with a migration background to check whether the sample was representative. Ultimately, 95% confidence intervals (CI) of participants characteristics were compared to actual proportions of the general Dutch population with a migration background.

Differences in movement behavior (activities/postures and PA) between patients with SCD and healthy adults were tested with the Mann-Whitney U test. Multivariable linear regression was performed for every posture/activity, with posture/activity as a dependent variable expressed in absolute hours or minutes per day. Independent variables included group plus season, gender, and age as confounders. For not normally distributed residuals, natural log transformations were used and back-transformed to enhance interpretability [36]. Differences in movement behavior between subtypes of SCD were similarly evaluated. Patients with HbSS were clustered with HbSβ^0^ and compared with HbSC clustered with HbSβ^+^ [2,37].

Associations between movement behavior and hemoglobin level, presence of avascular necrosis and NTproBNP level were evaluated by multivariable linear regression analysis, for every posture/activity as dependent variable. Season, gender, and age were included as confounders.

To account for differences in wear time, a sensitivity analysis was performed with activities and postures as percentage of wear time. To calculate wear time, spending 90 minutes or more lying down/non-wear was classified as non-wear. Checking for confounding was done with the 10% rule. The significance level was set at P < 0.05.

## Results

### Participant characteristics

A total of 36 patients with SCD signed informed consent and received an Activ8, of which 30 had sufficient PA data (defined as ≥4 days with at least 10 hours wear time per day). Ultimately, 57 healthy adults and 30 patients with SCD were included in the analysis. We did not encounter any other missing data. Participant characteristics are shown in Table 1. Healthy controls were older than patients with SCD (median age (IQR) 37.0 (30–50) vs. 32.1 (25–47) years respectively; P < 0.05). Seventeen patients (57%) had HbSS/HbSβ^0 and^ and 13 patients (43%) had HbSC/ HbSβ^+^. The median number of VOC/year was 0.5 (IQR 0–2). The median hemoglobin level in patients with SCD was 9.8 g/dL (IQR 8.4–10.8). Demographic characteristics of healthy controls correspond well to the general Dutch population with a migration background. The characteristics of healthy adults compared to the Dutch population are shown in S1 Table.

**Table 1:** Participant characteristics.

Variables	Patients with SCD (n = 30)	Healthy adults (n = 57)	P-value*
**Age (years)**			
Median (IQR)	32.1 (25-47)	37.0 (30-50)	0.026**
**Sex**			
Males, n (%)	13 (43.3)	31 (54.4)	0.230
**SCD genotype**			
HbSS, n (%)	15 (50.0)	N.A.	N.A.
HbSC, n (%)	8 (26.7)		
HbSβ + , n (%)	5 (16.7)		
HbSβ0, n (%)	2 (6.7)		
**VOC (admissions last year)**			
Median (IQR)	0.5 (0-2)	N.A.	N.A.
**Hydroxyurea**			
n (%)	13 (43.3)	N.A.	N.A.
**Hemoglobin level (g/dL)**			
Median (IQR)	9.8 (8.4-10.8)	N.A.	N.A.
**Activ8 wearing**			
n (%) 7 days weartime^a^	22 (73.0)	46 (80.7)	0.381
Median (IQR) hours daily weartime	16.32 (14.9-17.0)	16.3 (15.3-17.0)	0.651
**Season** ^**b**^			
n (%)	15 (50.0)	34 (59.6)	0.328
**Avasculair osteonecrosis, n (%)** **NTproBNP (ng/L)**	5 (17.0)		
Median (IQR)	50 (50-116)		
**BMI (kg/m**^**2**^)	22.6 (21-26)		

SCD = sickle cell disease, IQR = interquartile range, HbSS = hemoglobin-SS, HbSC = hemoglobin-C, HbSβ+ = hemoglobin sickle beta-plus thalassemia, HbSβ0 = hemoglobin sickle beta-zero thalassemia, VOC = vaso-occlusive crises, N.A. = not applicable.

^a^ Proportion of participants who wore the Activ8 during the instructed seven days

^b^ Proportion participants that wore the Activ8 in winter and autumn (vs summer/spring).

* P-value for any difference between groups, calculated with Mann-Whitney test for age and the chi-squared test for sex, Activ8 wearing and season.

** significant.

### Time spent on activities and postures

**Fig 1:**
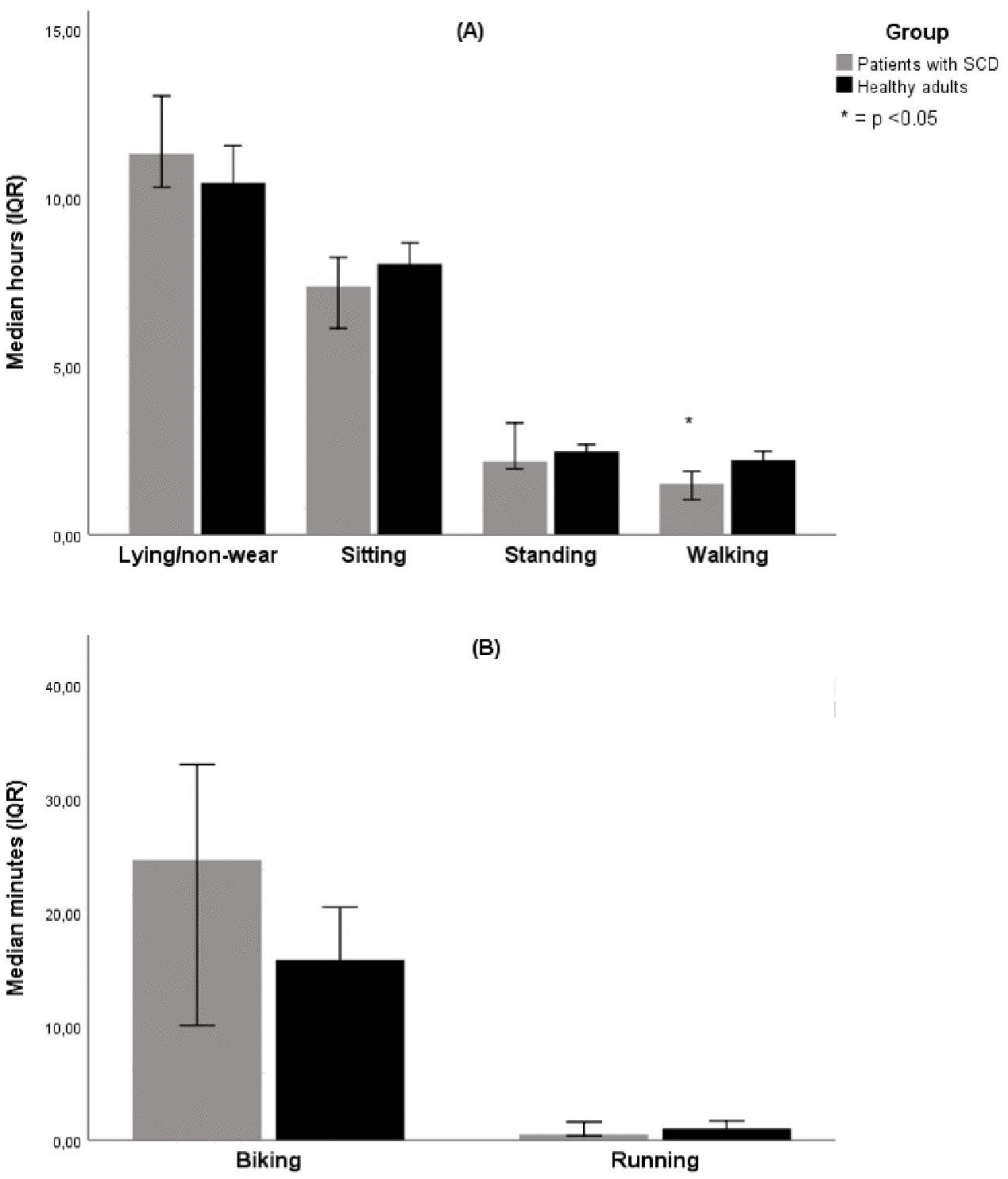
A. B Time spent in activities and postures per day. Mann-Whitney U test was used to compare groups.

Linear regression analysis using the different activities/postures as dependent variable and group plus confounders as independent variables are shown in Table 2. After adjusting for age, sex and season, linear regression showed that patients with SCD walk 0.68 (−1.22 to −0.14) hour/day (equivalent to approximately 3500–4000 steps) less than healthy controls. Crude differences in activities and postures are shown in Figs 1A, B and Table 2.

**Table 2:** Time spent on activities and postures. Linear regression analysis with activity as outcome and patients with SCD compared to healthy adults as determinant.

Activity or posture^a^	Patients with SCD	Healthy controls	Crude difference B [95%CI]	Adjusted difference B [95% CI]^b^	% B change	SE
Lying/non-wear (h/d)	12.0 (2.3)	10.7 (1.9)	1.25 [0.34–2.16]^c^	1.11 [−0.15 to 2.07]	−11.2	0.66
Sitting (h/d)	7.5 (1.8)	8.1 (1.7)	−0.54 [−1.31 to 0.22]	−0.62 [−1.44 to 0.20]	14.8	0.56
Standing (h/d)	2.5 (1.1)	2.5 (0.9)	−0.04 [−0.43 to 0.43]^d^	0.14 [−0.31 to 0.58]^d^	250	0.29
Walking (h/d)	1.6 (0.7)	2.3 (0.9)	−0.73 [−1.11 to −0.35]^c,d^	−0.68 [−1.07 to −0.28]^c,d^	−3.65	0.27
Biking (min/d)	24.4 (17.4)	20.4 (22.1)	3.95 [−5.25 to 13.15]^d^	5.37 [−4.21 to 14.94]^d^	35.95	6.54
Running (min/d)	1.3 (2.0)	4.1 (7.5)	−2.75 [−5.53 to 0.05]^d^	−2.34 [−5.28 to 0.60]^d^	−14.91	2.01

CI = confidence interval; SE=standard error

Interpretation: patients with sickle cell disease walk 0,68 hour per day less than healthy adults (adjusted for age, season, sex and multiple testing).

^a^ Time spent on activities and postures is reported in means (SD).

^b^ Adjusted for multiple testing using a Bonferroni correction, resulted in 99.2% CI and adjusted for age, sex and season.

^c^ significant.

^d^ Logarithmic transformations were performed to adjust for skewed residuals and back transformed to enhance interpretability

Individuals with HbSS and HbSβ^0^ (n = 17) walked 0.47 (CI −1.01 to 0.06) hour/day (equivalent to approximately 3000 steps) less than those with HbSC and HbSβ^+^ (n = 13), adjusted for age, sex and season. No differences were identified for lying/non-wear, sitting, standing, biking and running. Crude differences and multivariable linear regression analysis using the different activities/postures as dependent variables and group plus confounders as independent variables are shown in S2 Table. When hemoglobin level was added to the model the differences in walking duration between patients with HbSS/HbSβ^0^ and HbSC/ HbSβ^+^decreased to 0.32 (CI-1.01 to 0.38), suggesting that hemoglobin level partly explained the differences between the subgroups.

Multivariable linear regression, adjusted for sex, age and season, showed an increase in walking duration of 0.26 hours (CI 0.01 to 0.52) for every point increase in hemoglobin level.

No significant associations between the presence of avascular osteonecrosis or NTproBNP levels and activities and postures were identified.

### Sensitivity analysis

To account for differences in wear time, absolute time spent on activities and postures was expressed as a percentage of total wear time. Linear regression, adjusted for age, sex, season and multiple testing, identified differences between patients with SCD and healthy controls in lying/non-wear (SCD 21.2 ± 6.7 vs healthy controls 15.6 ± 6.7, B 5.0 99.2%CI [0.2–9.8]) and walking (SCD 10.0 ± 3.8 vs healthy controls 14.2 ± 5.1, B −3.99 99.2%CI [−7.1 to −0.9]). Linear regression analysis of the other postures/activities as percentage of wear time are presented in S3 Table.

### Physical activity during the day

**Fig 2:**
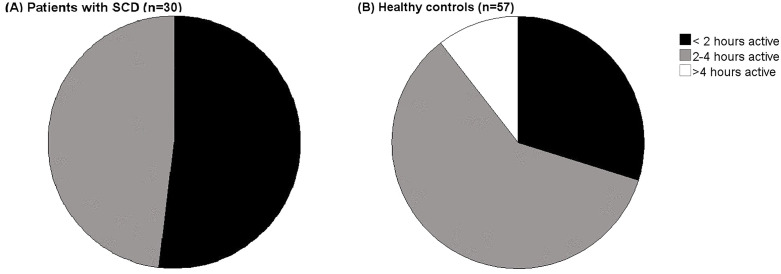
A. B Distribution of activity hours per day across three categories, expressed as percentages, for patients with SCD and healthy adults.

Compared to healthy adults, patients with SCD were significantly less physically active during the day (defined as the combined time spent on walking, biking and running) (SCD 2.0 ± 0.8 vs healthy controls 2.7 ± 1.0 p < 0.05). Figs 2A, B illustrate the distribution of activity hours per day across three categories (in percentage) for both groups.

## Discussion

We compared movement behavior of patients with SCD in the Netherlands with healthy controls with a migration background, using an objective measurement tool, to better understand movement behavior in this population. Patients with SCD walked 40 minutes per day less than healthy controls. A trend was observed for individuals with HbSS or HbSβ^0^ walking 30 minutes per day less than patients with HbSC or Hbsβ^+^. No differences were identified as compared to controls or between patient groups for other postures and activities such as biking and running. A higher hemoglobin level was associated with an increase in walking duration. We did not identify associations with avascular osteonecrosis or NTproBNP levels.

Results of the current study are in accordance with a previous study evaluating PA and SB in children and adolescents with SCD, which showed lower levels of moderate and vigorous PA compared to healthy controls [22]. Other studies evaluating movement behavior in non-oncological hematological diseases, like haemophilia and thalassemia, show the same trend of lower PA levels [25,38]. Reduced PA in patients with SCD seems partly related to the degree of anemia. Other factors that may influence PA in this population are pain, negative personal beliefs about PA on disease impact and kinesiophobia [10,23,39]. These factors may discourage PA and restrict activity levels during the day.

A key strength of this study is the use of a validated accelerometer to objectively assess movement behavior, which is a more precise method than self-reported PA [40]. Furthermore, we carried out a sensitivity analysis to eliminate differences in wear time to analyse time spent on activities/postures as percentage of wear time. The Activ8 is able to identify postures and activities, unlike most accelerometers or questionnaires that report movement behavior variables in Metabolic Equivalent of Task (METs), counts or steps, which are sometimes challenging to interpret. This study improves the interpretation by reporting different postures and activities in hours or minutes per day. To our knowledge, this is the first study objectively assessing movement behavior in adults with SCD, providing practice-relevant insights.

The main limitation of this study is the relative small sample size in the SCD group. Given the considerable variation, mainly in standing, biking and running, the sample size of patients with SCD might have been too small to detect significant differences as compared to controls or between patient groups. Moreover, the small proportion of patients with avascular osteonecrosis might have impaired the ability to identify an association with movement behavior. Similarly, little variation and relatively low NTProBNP levels could have impeded the ability to identify an association with movement behaviour as well. Lastly, a relatively small sample size may limit generalizability and thus external validity, although the representation in genotypes in the current study is similar to the Dutch population of SCD patients with exception of the relative large group of HbSβ+ patients in this study [35]. Generalizability to countries with a higher prevalence of SCD is limited due to differences in medical treatment availability. As SCD is a rare disease, with a prevalence of 1250–1500 in the Netherlands, the challenge of small sample sizes is inherent to SCD [7]. A second limitation of the current study is the inability of the Activ8 to distinguish between lying down and non-wear time. Therefore, the cumulative time registered as lying down might be confounded by non-wearing time. Likewise, time spent in activities such as swimming or other sports activities that required removal of the Activ8, could not be measured. This could potentially underestimate the real PA time. Moreover, we were unable to differentiate between biking with or without electric support is. It is expected that this did not influence the results of the current study, as biking was rarely performed in all of the included groups.

Patients with SCD walk an average of 40 min per day, equivalent to 3500–4000 steps, less compared to healthy adults. These differences in movement behavior seem especially relevant for patients with SCD, because even small changes in movement behavior can lead to clinically meaningful health benefits. An increase of just 500 steps/day has been identified as the minimal clinically important difference (MCID), associated with 8–9% reduction in the risk of all-cause mortality in active adults [41]. In other chronic diseases, including COPD, asthma and Parkinson Disease the MCID ranges from 400–1400 steps/day. [42–44]. Moreover, a higher daily step count is a significant predictor of lower scores on self-reported pain in activities of daily living, physical functioning and depressive symptoms in patients with fibromyalgia [45]. Given the negative personal beliefs about disease impact on PA in SCD, it is important to underline that even minor differences in movement behavior may impact daily life of patients with SCD [23].

The high daily sitting time may be relevant for patients with SCD as this is part of SB, which is associated with adverse health effects in different chronic diseases similar to SCD and is an important risk factor for all cause mortality [18,46,47]. Although we did not find differences between patients with SCD and controls with the same migration background in sitting time, the rather high levels of 7.5 h/d sitting by patients with SCD may suggest a risk for unfavorable outcomes. The high amount of time spent on sedentary activities and short time of biking and running observed in both groups indicates that movement behavior is not only explained by disease specific factors and healthy controls may not serve as an ideal benchmark. Given that 50% of the Dutch population fails to meet the physical activity guidelines, the even lower PA levels in patients with SCD may be a risk for adverse health outcomes [48]. Specific interventions directed at behavioural change to reduce sedentary time may therefore be considered for patients with SCD, as reducing sedentary time requires a different approach than increasing physical activity. [49].

Many individuals with SCD are not aware of the safety and benefits of engaging in PA [50]. Physiotherapy could play a key role in supporting patients with SCD to improve physical literacy (i.e., the motivation, confidence, physical competence, knowledge, and understanding to value and take responsibility for engagement in physical activities for life) [51]. Given the heterogeneity of symptoms in SCD, an individual approach is necessary to improve movement behavior and should be structurally integrated in the multidisciplinary care of patients with SCD.

As hemoglobin levels only partly explains differences in movement behavior in patients with SCD, future studies should focus on identifying additional factors that can explain differences in movement behavior in SCD. This should be approached from a biopsychosocial model, including signs of organ damage, pain, fatigue, and personal beliefs and knowledge about physical activity. Identifying these factors could help to predict which patients are at risk for further reductions in physical activity, which has been associated with a higher risk of mortality in in the general population and many chronic diseases. Besides the potentially long term effect of being physically active for patients with SCD, increasing PA and, particularly, walking (light to moderate PA) may positively affect physical functioning [10]. Therefore, future studies should focus on identifying which activities or physical training would be well tolerated and yield the most health benefits for patients with SCD. Moreover, future studies should aim for a multicentre approach, possibly even international, in order to increase the possible sample size and improve generalizability of the results. When studying movement behaviour, an activity diary could be added to the accelerometer measurement to prevent misclassification during non-wear of the accelerometer.

In conclusion, movement behavior in adults with SCD differs from healthy controls, which is mainly reflected by reduced walking during the day. No differences were identified for other postures and activities. Currently, physical activity is not structurally addressed in SCD care pathways. This study highlights a potential gap in supportive care. Tailored guidance aimed at positively changing movement behavior is needed in this population, and physical therapists could play a key role in facilitating this improvement in activity levels.

## Supporting information

S1 Table Characteristics of the study cohort of healthy adults with migration background compared to the Dutch population with migration background. (DOCX)

S2 Table Linear regression analysis with activity as outcome and patients with HbSS/ HbSβ^0^ compared to HbSC/HbSβ^+^ as determinant. (DOCX)

S3 Table Time spent on activities and postures as percentage of wear time. Linear regression analysis with activity as outcome and patients with SCD compared to healthy adults as determinant.
(DOCX)
