# Supplementary material for: Movement behavior in adults with sickle cell disease compared to healthy adults: a cross-sectional study
Source: PLoS One. 2026 Apr 15;21(4):e0336932. doi: 10.1371/journal.pone.0336932 (PMC13082650; doi:10.1371/journal.pone.0336932)
Supplement: S1 Table — (DOCX) [file pone.0336932.s001.docx]

Supplemental table 1. Characteristics of the study cohort of healthy adults with migration background compared to the Dutch population with migration background.

| Variables^a^ | Healthy adults with migration background % [95% CI] | Dutch population with migration background % |
| --- | --- | --- |
| Education level |  |  |
| Low | 21 [12 – 33] | 29 |
| Middle | 35 [24 – 48] | 34 |
| High | 44 [32 – 57] | 37 |
| Living area |  |  |
| Urban | 88 [77 – 94] | 84 |
| Suburban | 5 [2 – 13] | 1^b^ |
| Rural | 7 [2 – 15] | 14 |
| Morbidity |  |  |
| ≥ 1-long term condition | 21 [10 – 32] | 32 |

CI = confidence interval.

^a^Definitions from Statistics Netherlands (CBS) were used^33^.

^b^value was not captured by 95% CI.
