# Supplementary material for: Movement behavior in adults with sickle cell disease compared to healthy adults: a cross-sectional study
Source: PLoS One. 2026 Apr 15;21(4):e0336932. doi: 10.1371/journal.pone.0336932 (PMC13082650; doi:10.1371/journal.pone.0336932)
Supplement: S2 Table — (DOCX) [file pone.0336932.s002.docx]

Supplemental table 2. Linear regression analysis with activity as outcome and patients with HbSS/ HbSβ^0^ compared to HbSC/HbSβ^+^ as determinant.

| Activity or posture^a^ | HbSS/ HbSβ^0^ | HbSC/ HbSβ^+^ | Crude difference B [95%CI] | Adjusted difference B [95% CI] |
| --- | --- | --- | --- | --- |
| Lying/non-wear (h/d) | 11.5 (10.0-14.2) | 11.3 (10.0-12.5) | 0.81 [-0.90 to 2.51] | 0.44 [-1.25 to 2.13] |
| Sitting (h/d) | 7.2 (5.9-9.0) | 7.3 (6.0-8.3) | 0.40 [-0.96 to 1.75] | 0.67 [-0.73 to 2.08] |
| Standing (h/d) | 2.1 (1.6-2.7) | 3.3 (2.2-3.7) | -0.64 [-1.43 to 0.16] | -0.56 [-1.39 to 0.27] |
| Walking (h/d) | 1.5 (0.9-1.9) | 1.8 (1.3-2.4) | -0.44 [-0.93 to -0.05] | -0.47 [-1.01 to 0.06] |
| Biking (min/d) | 24.7 (7.8-32.7) | 28.6 (9.8-43.5) | -0.14 [-0.35 to 0.08] | -0.09 [-0.30 to 0.13] |
| Running (min/d) | 0.4 (0.1-0.9) | 1.1 (0.4-2.1) | <0.01 [-0.03 to 0.02] | <0.01 [-0.03 to 0.03] |

CI = confidence interval
^a^ Time spent on activities and postures is reported in means (SD).

^b^ Adjusted for age, season, sex
Logarithmic transformations were performed to adjust for skewed residuals and back transformed to enhance interpretability
