# Supplementary material for: Movement behavior in adults with sickle cell disease compared to healthy adults: a cross-sectional study
Source: PLoS One. 2026 Apr 15;21(4):e0336932. doi: 10.1371/journal.pone.0336932 (PMC13082650; doi:10.1371/journal.pone.0336932)
Supplement: S3 Table — Linear regression analysis with activity as outcome and patients with SCD compared to healthy adults as determinant. (DOCX) [file pone.0336932.s003.docx]

Supplemental table 3. Time spent on activities and postures as percentage of wear time. Linear regression analysis with activity as outcome and patients with SCD compared to healthy adults as determinant.

| Activity or posture^a^ | People with SCD | Healthy adults | Crude difference B [95%CI] | Adjusted difference B [99.2% CI]^b^ |
| --- | --- | --- | --- | --- |
| Lying/non-wear (h/d) | 19.02 | 13.58 | 5.58 [2.30 to 8.86] | 4.99 [0.22 to 9.77] |
| Sitting (h/d) | 46.28 | 49.81 | -3.00 [-7.00 to -1.01] | -3.45 [-9.19 to 2.29] |
| Standing (h/d) | 14.38 | 16.02 | -0.06 [-2.58 to 2.46] | 0.64 [-2.95 to 4.23]^d^ |
| Walking (h/d) | 10.51 | 10.02 | -4.14 [-6.29 to -1.99] | -3.99 [-7.06 to -0.91]^c,d^ |
| Biking (min/d) | 2.53 | 1.66 | 0.41 [-0.53 to 1.34] | 0.5 [-0.78 to 1.88]^d^ |
| Running (min/d) | 0.05 | 0.14 | -0.27 [-0.56 to 0.02] | 0.07 [-0.32 to 0.45]^d^ |

CI = confidence interval
^a^ Time spent on activities and postures is reported in means (SD).
^b^ Adjusted for multiple testing using a Bonferroni correction, resulted in 99.2% CI and adjusted for age, sex and season.
^c^ significant.
^d^ Logarithmic transformations were performed to adjust for skewed residuals and back transformed to enhance interpretability
